# Pyromellitic acid grafted to cross-linked LDH by dendritic units: An efficient and recyclable heterogeneous catalyst for green synthesis of 2,3-dihydro quinazoline and dihydropyrimidinones derivatives

**DOI:** 10.1016/j.heliyon.2023.e20978

**Published:** 2023-10-17

**Authors:** Nastaran Ghanbari, Hossein Ghafuri

**Affiliations:** Catalysts and Organic Synthesis Research Laboratory, Department of Chemistry, Iran University of Science and Technology, Tehran, 16846-13114, Iran

**Keywords:** Layered double hydroxide (LDH), Pyromellitic acid (PMA), Organic-inorganic catalyst, 2,3-Dihydro quinazoline derivatives, 3,4-Dihydropyrimidinone-2-(1*H*)-Ones derivatives

## Abstract

In this work, using layered double hydroxide (LDH) inorganic substrate, melamine as binding agent and dendrimer G1 and also pyromellitic acid (PMA) organic catalytic agent a heterogeneous acid catalyst was designed and prepared. After that, the prepared organic-inorganic catalyst was evaluated by various identification techniques such as FTIR, EDX, XRD, TGA, FESEM, and BET, and the results showed that the desired structure was successfully prepared. Also, in order to investigate the efficiency of the LDH@Me-PMA nanocatalyst as an efficient and heterogeneous catalyst, it was used for green and one-pot synthesis of 2,3-dihydro quinazoline and 3,4-dihydropyrimidinone-2-(1H)-ones derivatives. The use of LDH@Me-PMA catalyst led to the synthesis of the desired derivatives with higher efficiency and shorter reaction time than previously reported works. In addition, the prepared LDH@Me-PMA acid catalyst has the ability to be recycled and reused for 5 consecutive periods and has high stability, which is well consistent with the principles of green chemistry.

## 1 Introduction

Dendrimers are known as a new class of polymeric materials [1,2]. Dendrimers are molecules with a radially symmetrical structure and nanometer size, which have a well-defined and homogeneous structure [[3], [4], [5], [6], [7]]. They also have tree-like arms or branches. These branched molecules were first discovered in 1978. They are known only as an architectural motif, not a compound [8,9]. Also, dendrimers are multi-branched structures that have a precise architecture, their end groups can be functionalized, thus modifying their physical, chemical, or biological properties [10]. These unique properties of dendrimers have led them to be of special interest for a wide range of industrial and medical applications [11]. Therefore, by placing catalytic agents in the outer part of the dendrimer, it can be used as a heterogeneous catalyst in organic reactions [12]. The catalytic agents placed in the dendrimer enable the control of the microenvironment around these catalytic agents [13,14]. In the event that placing a single catalytic group in the large dendrimer results in a low-loaded system. One of the ways to solve this problem is to combine catalytic groups throughout the dendrimer [15]. Catalytic groups on the surface of dendrimers are easily accessible in reactions, especially in large dendrimers that have a spherical composition with end groups on the surface [16,17]. In addition, the amount of loading of catalytic groups on the terminal part of dendrimers is very high due to the inherent nature of their structure [18]. There are many examples of the use of dendrimers functionalized by catalytic groups as heterogeneous catalysts in organic reactions such as Heck couplings [19], oxidation [20], decarboxylation [21], alkyl alkylation [22], Knoevenagel condensation [23], etc.

Melamine is a white solid trimer of cyanamide, with a 1,3,5-triazine skeleton. Similar to cyanamide, it contains 67 % nitrogen in mass and its derivatives have fire-retardant properties due to the release of nitrogen gas when burning or charring [24,25]. Also, it is used as dendrimer G1 for catalyst synthesis.

Benzene-1,2,4,5-tetracarboxylic acid (Pyromellitic acid (PMA)) is a group of benzene polycarboxylic acids that has been well considered as an acidic compound for the synthesis of catalysts and adsorbents [26]. Also, it has found applications in creating hydrogen bond arrays and especially in coordination synthesis to prepare polymers and mixed ligand systems.

Layered double hydroxides (LDHs) are a class of ionic layered compounds consisting of positively charged brucite-like layers with an interlayer region containing solvent, water molecules, and anions [[27], [28], [29], [30], [31]]. LDHs usually contain divalent and trivalent metal cations, which have the general formula [M^2+^_(1-x)_ M^3+^_(x)_ (OH)_2_]^x +^ A^n-^_x/n_. yH_2_O where M^2+^ is a divalent metal, M^3+^ is a trivalent metal, and A^n−^ is a charge-compensating inorganic or organic anion [32]. Today, the physical and chemical properties of LDHs have been widely studied; Therefore, these materials have been widely used as precursors for the preparation of adsorbents [28,33], heterogeneous catalysts [34], ion exchange [35], polymer/LDH nanocomposites [36], and drug delivery [37].

The use of efficient catalysts instead of stoichiometric processes is one of the main requirements of environmentally friendly and sustainable chemistry [38]. Among known catalytic reactions, reactions catalyzed by heterogeneous catalysts are one of the important principles in modern organic chemistry and are of great importance for various applications such as chemical industries, biochemical, and pharmaceutical industry [[39], [40], [41]]. Therefore, the use of homogeneous catalysts on a large scale, especially in the synthesis of pharmaceutically active substances [42,43], creates serious problems in the final product. Hence, the immobilization of catalytic agents on heterogeneous and recyclable substrates for easy separation and reuse of the catalyst is an important and key principle for the development of green chemistry and sustainable [44,45]. The recyclability and easy separation of heterogeneous catalysts is an important issue from the point of view of green chemistry as well as from the economic point of view [46]. Catalysts based on layered double hydroxides provide easy separation of the catalyst from the reaction.

One of the most obvious features of nanotechnology is its potential use in various fields. Nanomaterials have unique properties that cannot be seen in bulk materials. The discovery of nanoparticles with different size, shape, and composition has attracted significant opinions of scientists in different fields [47]. Due to their specific surface area, nanostructures have shown the potential to respond to the interface between homo and heterogeneous catalysts. In nanocatalysts, the word “nano” refers to very small particles in nanoscale size [48]. Since nanoparticles have special properties such as a large surface-to-volume ratio compared to other materials, they are a suitable alternative to conventional catalysts [49]. Nanocatalysts, which consist of one or more materials with catalytic properties that have at least one nanoscale dimension, are rapidly developing. Nanocatalysis can help design catalysts with excellent activity, greater selectivity, and high stability. These characteristics can easily be achieved by tailoring the size, shape, morphology, composition, electronic structure, and thermal and chemical stability of the particular nanomaterial [50,51].

Nitrogen-containing heterocyclic rings exist in various compounds such as drugs, dyes, organic substances, and especially in biologically active molecules [[52], [53], [54], [55]]. In recent decades, special attention has been paid to the development of heteroaromatic organic compounds, such as benzothiazoles [56], acridine [57,58], benzimidazoles [59], quinolones [[60], [61], [62]], and triazoles [63]. Derivatives of 2,3-dihydro quinazoline are an important category of heterocyclic compounds that have a wide range of biological activity and medicinal properties [64]. They can also be easily oxidized to their biologically active analogs. There are various methods for the synthesis of 2,3-dihydro quinazoline derivatives, and the use of a catalyst is the best and most practical method [65,66]. Also, in this direction, various catalysts such as ionic liquids [67], and Fe_3_O_4_ nanoparticles [68] have been used for the synthesis of,3-dihydro quinazoline derivatives.

Derivatives of dihydropyrimidinones as heterocycle compounds containing nitrogen have attracted special attention due to their therapeutic and biological activities [69,70]. Also, derivatives of dihydropyrimidinones have wide applications in the field of calcium channel blockers, antihypertensive, and neuropeptide Y antagonists [71]. These compounds were prepared more than a hundred years ago using a three-component, one-pot protocol with ketoester, aldehyde, and urea under acidic conditions. This method of preparing dihydropyrimidinones derivatives due to low efficiency led to the improvement of the method and its modification by using different catalysts such as poly phosphate ester (PPE) [72], FeCl_3_ [73], TsOH [74], zeolite [75], and KSF montmorillonite [76].

**Scheme 1:**
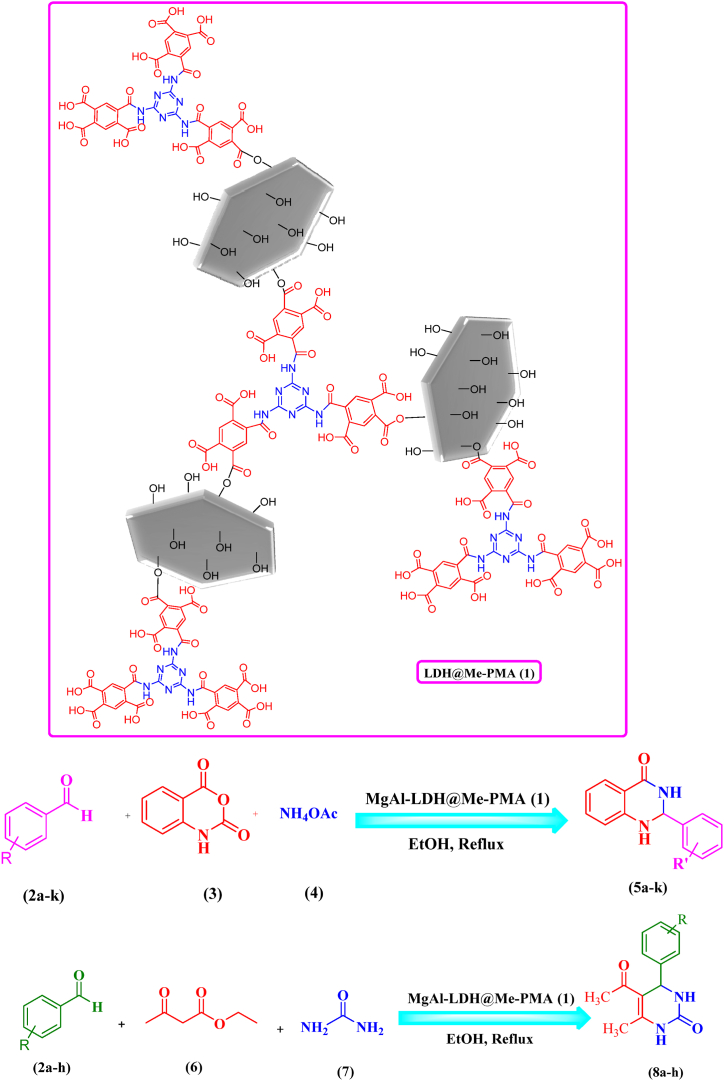
LDH@Me-PMA (**1**) as catalyst for green synthesis of 2,3-dihydro quinazoline and 3,4-dihydropyrimidinone-2-(1*H*)-ones derivatives in EtOH solvent under reflux conditions.

Based on the contents mentioned above, in this work, we prepared an acidic organic-inorganic heterogeneous catalyst LDH@Me-PMA (**1**) by using Mg–Al LDH inorganic substrate, melamine as dendrimer G1 (Me), and pyromellitic acid (PMA) in a green and easy process using available and cheap materials (Scheme 1). Also, after identifying its structure through different analyses, we used it as an efficient and recyclable heterogeneous catalyst to synthesis 2,3-dihydro quinazoline and dihydropyrimidinones derivatives with high efficiency. Compared to previously reported works, this prepared catalyst has advantages such as green synthesis, easy and cheap method, use of available materials, and ability to recycle the heterogeneous catalyst and reuse it in subsequent reactions.

## 2 Experimental

### 2.1 Reagents and apparatus

The chemicals were purchased from Aldrich or Merck with high purity and used in experiments without purification. LDH@Me-PMA nanocomposite (**1**) using analysis FTIR (Shimadzu 8400s), EDX (Numerix DXP-X10P), XRD patterns (TW 1800 diffractometer with CuKα radiation (λ = 1.54050 Å)), FESEM (TESCAN-MIRA3), BET (ASAP 2020 micromeritics), and TGA (Bahr Company STA 504) were examined.

### 2.2 Preparation method of Mg–Al LDH

Mg–Al LDH was prepared using previously reported methods [28]. First, in a 250 mL flask, 100 mL of 3 M urea aqueous solution was stirred, then 5.13 g of Mg(NO_3_)_2_⋅6H_2_O and 3.75 g of Al(NO_3_)_3_.9H_2_O were added. Next, the temperature increased to 100 °C, and the reaction mixture was stirred for 12 h. Then, the temperature was reduced to 94 °C, and the reaction aged for another 12 h. Finally, the created suspension was centrifuged and washed with deionized water several times, then dried at 85 °C for 12 h.

### 2.3 Preparation method of Me-PMA

First, pyromellitic acid (PMA, 1.5 g), hydroxybenzotriazole (HOBT, 0.8 g), and 1-Ethyl-3-(3-dimethylaminopropyl) carbodiimide (EDCI, 0.92 g) were dissolved in 10 mL acetonitrile and was stirred for 30 min at room temperature. In the following, melamine (Me, 0.25 g) was added and stirred for 24 h at room temperature. Then, the white precipitate was filtered, and washed with acetonitrile and EtOH, and dried at 85 °C for 12 h.

### 2.4 Preparation method of LDH@Me-PMA

**Scheme 2:**
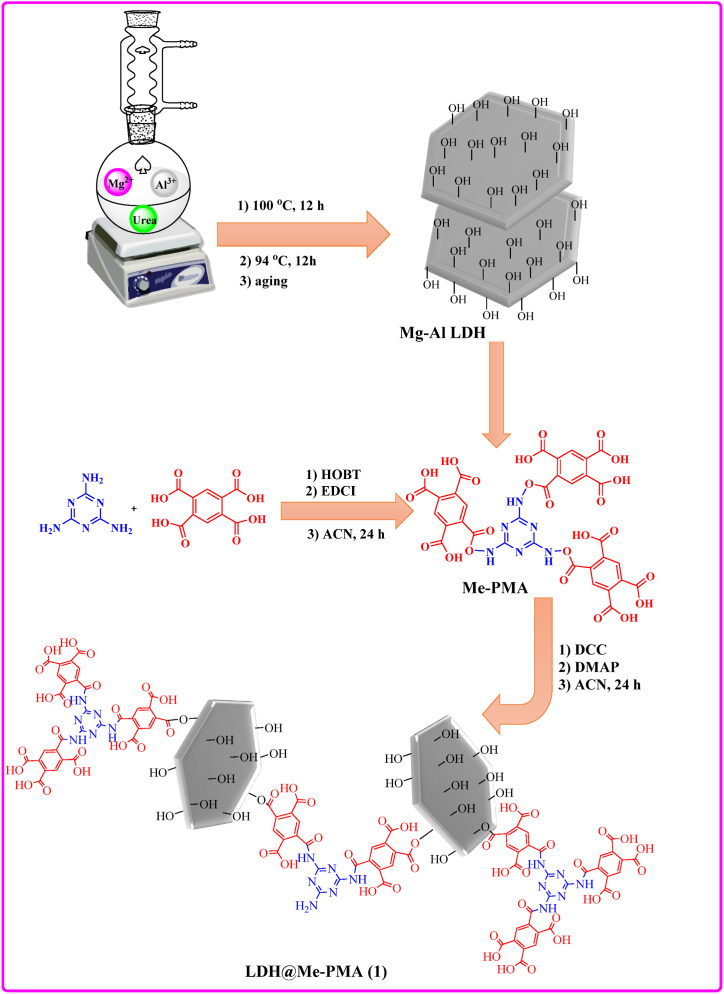
Schematic preparation of LDH@Me-PMA nanocomposite (**1**).

Mg–Al LDH (0.5 g) was dissolved in acetonitrile (10 mL), then, dicyclohexylcarbo-diimide (DCC, 0.28 g) and 4-dimethylaminopyridine (DMAP, 0.16 g) were added and stirred for 1 h. Next, Me-PMA (0.5 g) was added and stirred at room temperature for 24 h. Then, the mixture was filtrated and washed with acetonitrile and EtOH. Finally, the precipitate was dried at 80 °C for 12 h (Scheme 2).

### 2.5 General procedure synthesis of 2,3-dihydroquinazoline derivatives

A mixture of aldehyde (**2**, 0.5 mmol), isatoic anhydride (**3**, 0.5 mmol), ammonium acetate (**4,** 2.5 mmol), and LDH@Me-PMA (**1**, 30 mg), in EtOH (5 mL) was stirred under reflux conditions. After completion of the reaction, the heterogeneous catalyst was separated by filtration and washed with EtOH. Then, the reaction mixture was allowed to cool and 5 mL of distilled water was added to obtain the desired pure crystals of 2,3- dihydroquinazoline derivative. The recovered catalyst was suspended in acetone (3 mL) and stirred at room temperature for 15 min. Then, the catalyst (**1**) was filtered and dried at 80 °C for 6 h to be used in the next reactions.

### 2.6 General procedure synthesis of 3,4-dihydropyrimidinone-2-(1H)-ones derivatives

A mixture of aldehydes (**2**, 1 mmol), ethyl acetoacetate (**6**, 1 mmol), urea (**7**, 1.2 mmol), and LDH@Me-PMA (**1**, 30 mg) in EtOH (5 mL) was stirred reflux conditions. Completion of the reaction was observed by TLC (n-hexane: EtOAc, 3:1). After completion of the reaction heterogeneous catalyst was separated by filtration. Then, the reaction mixture was allowed to cool and 5 mL of distilled water was added to obtain the desired pure crystals of 3,4-dihydropyrimidinone. The recovered catalyst was suspended in acetone (3 mL) and stirred at room temperature for 15 min. Then, the catalyst (**1**) was filtered and dried at 80 °C for 6 h to be used in the next reactions.

### 2.7 Measurement of the acidity of the LDH@Me-PMA nanocomposite (**1**) using back titration

The back titration method was used to determine the acidity of LDH@Me-PMA (**1**) fresh and after-recycling samples. Hence, distilled water (35.0 mL), NaCl (0.5 g), LDH@Me-PMA (**1**, 0.5 g), and aqueous NaOH (10.0 mL, 0.1 M) were added into a beaker and stirred at room temperature for 24 h. Then, a few drops of phenolphthalein (as an indicator) were added, and the excess amount of [OH^−^] was titrated with HCl (0.1 M) until the color changed from pink to colorless. Finally, the pH of LDH@Me-PMA (**1**) fresh and after-recycling were calculated which were 2.24 and 2.6, respectively.

## 3 Results and discussion

**Fig. 1:**
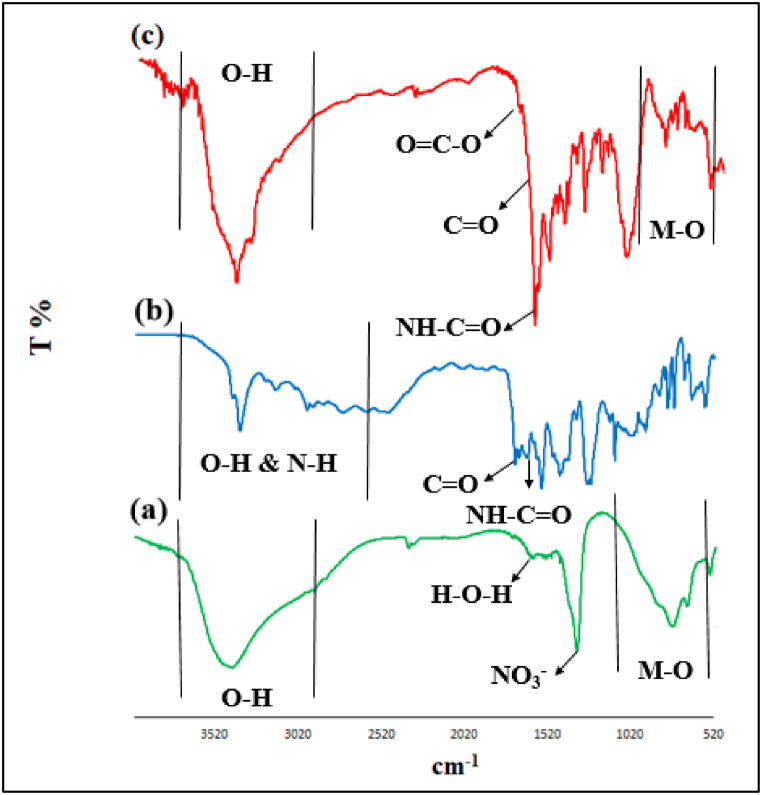
FTIR spectra of Mg–Al LDH (**a**), Me-PMA (**b**), and LDH@Me-PMA (**1**, **c**).

FTIR spectra of Mg–Al LDH (**a**), Me-PMA (**b**), and LDH@Me-PMA (**1**, **c**) are shown in Fig. 1. In the FTIR spectrum of Mg–Al LDH, the absorption band in the region of 3452 cm^−1^ is assigned to O–H groups. Also, the absorption bands in the region of 1616 cm^−1^ and 1354 cm^−1^ are related to the H–*O*–H bending vibrations of water molecules and nitrate anions between the layers, respectively. In addition, the absorption band at 900-560 cm^−1^ corresponds to Al–OH, Mg–O, and Al–O bonds (Fig. 1a).

Fig. 1b shows the FTIR spectra of Me-PMA. The broad absorption band at 3420-2900 cm^−1^ is related to N–H and O–H groups. Also, the absorption bands at 1719 cm^−1^ and 1643 cm^−1^ are related to the stretching vibrations of C=O in acid and amide groups, respectively.

FTIR spectrum of LDH@Me-PMA (**1**) is presented in Fig. 1c. The broad absorption band at 3420-2900 cm^−1^ is related to O–H groups. In addition, the absorption bands at 1735 cm^−1^, 1714 cm^−1^, and 1646 cm^−1^ belong to the stretching vibrations of C=O bond of ester, acid, and amide groups, respectively. Also, absorption bands at 500-900 cm^−1^ are related to Mg–O and Al–O vibrations.

**Fig. 2:**
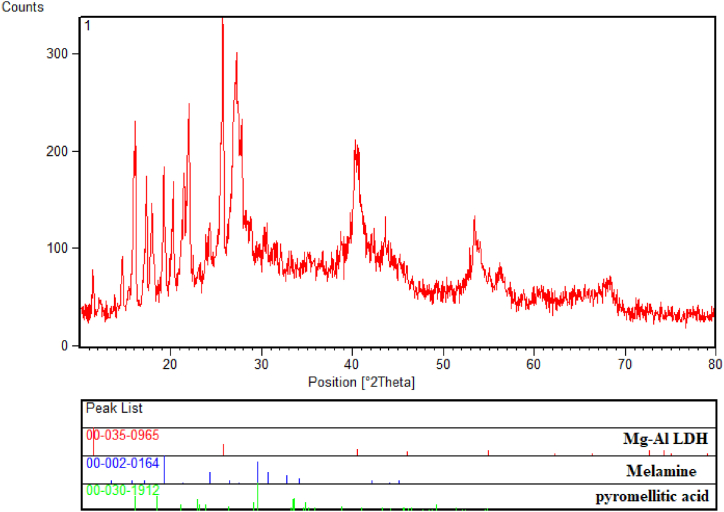
XRD patterns of LDH@Me-PMA nanocomposite (**1**).

The XRD pattern of LDH@Me-PMA nanocatalyst (**1**) is shown in Fig. 2. The peaks observed in the wide-angle XRD pattern are in good agreement with all three Joint Committee on Powder Diffraction Standards (JCPDS) card no Mg–Al LDH (00-035-0965), melamine (00-002-0164) and pyromellitic acid (00-030-1912). Moreover, symmetrical reflections at 2θ = 11, 15°, 23°, 17°, 19°, 21°, 23°, 29°, 41°, 45°, 54°, 58°, 62°, 69°, and 71° show the Successful preparation of LDH@Me-PMA nanocomposite (**1**).

**Fig. 3:**
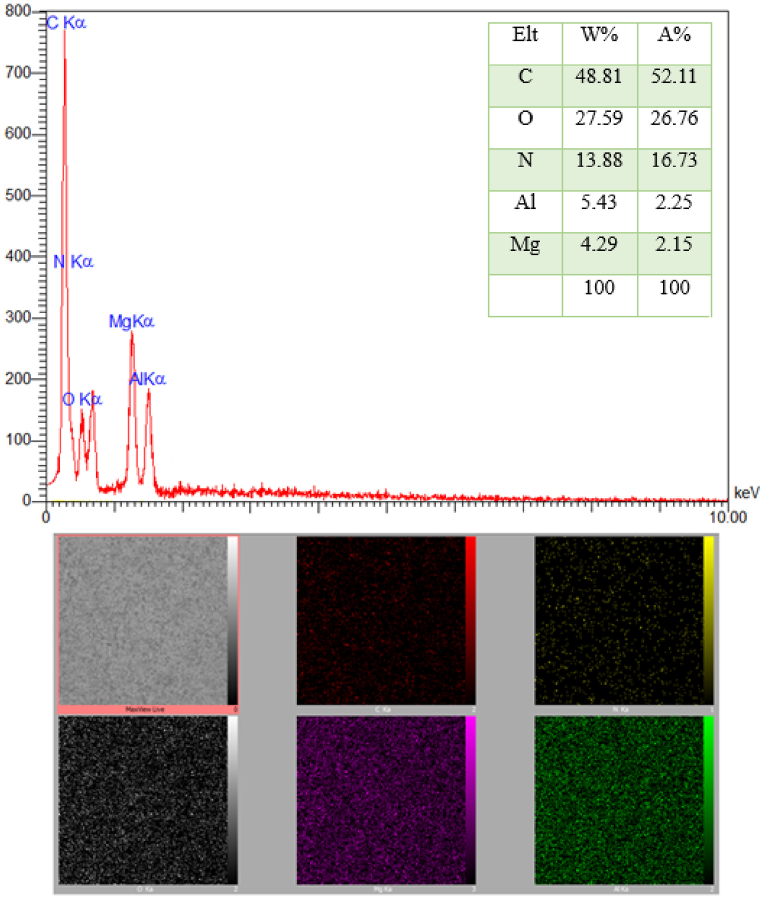
EDX spectra of LDH@Me-PMA nanocomposite (**1**).

EDX analysis and mapping images confirm the presence of elements C (48.81 %), O (27.59 %), N (13.88 %), Al (5.43 %), and Mg (4.29 %) in the nanocatalyst structure. Also, the mapping images show the uniform distribution of these elements Fig. 3.

**Fig. 4:**
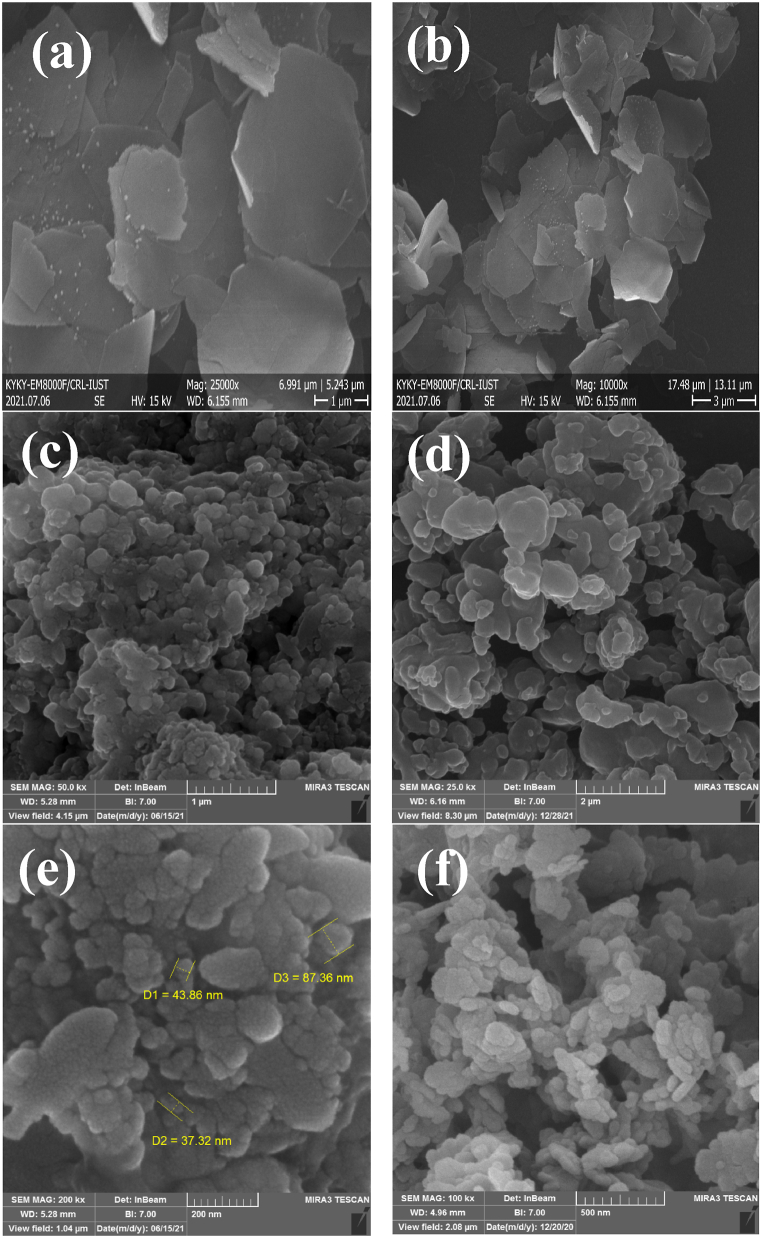
FESEM images of MgAl-LDH (**a** and **b**) and LDH@Me-PMA nanocomposites (**1, c-f**).

FESEM images of MgAl-LDH and LDH@Me-PMA nanocomposite (**1**) are shown in Fig. 4. As can be seen, images **a** and **b** show the morphology of the MgAl-LDH. Also, images **c-f** show the morphology of the LDH@Me-PMA nanocomposite (**1**). Based on these images, it can be clearly seen that the final structure is created in the form of irregular plates with a diameter of about 37–87 nm. This accumulation and irregularity in the plates indicate the placement of organic components between and on the LDH plates and confirm the formation of the final composite.

**Fig. 5:**
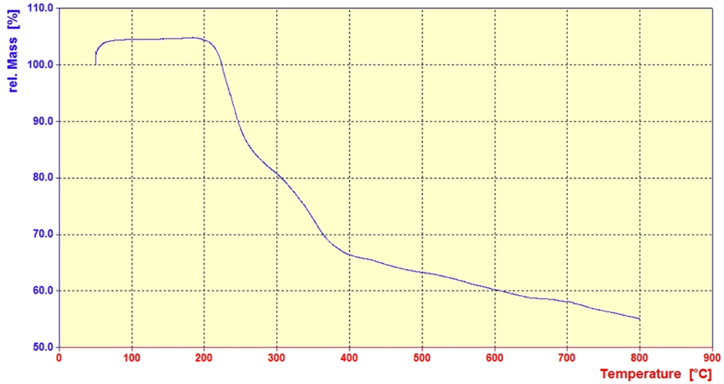
TGA curve of the LDH@Me-PMA nanocomposite (**1**).

TGA analysis related to thermal stability of LDH@Me-PMA nanocomposite (**1**) is presented in Fig. 5. The TGA curve shows two weight reductions in the region of 210–250 °C and 280–390 °C. hence, these two observed weight loss are related to the decomposition of the organic parts of the prepared composite structure. In addition, the curve from the temperature of 430 °C has a constant slope, which is related to the inorganic parts of Mg–Al LDH.

**Fig. 6:**
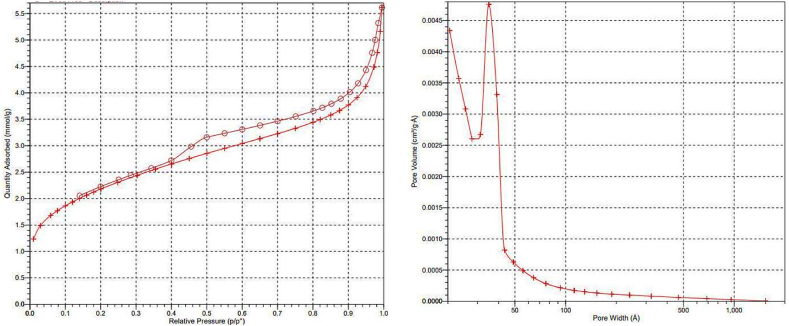
N_2_ adsorption–desorption isotherms of LDH@Me-PMA nanocomposite (**1**).

BET surface area and pore volume of LDH@Me-PMA nanocomposite (**1**) were calculated according to the N_2_ adsorption–desorption isotherm (Fig. 6). As can be observed, LDH@Me-PMA nanocomposite (**1**) showed BET surface area (170.22 m^2^/g) and pore volume (0.15 cm^3^/g).

## 4 Optimization of the reaction conditions using LDH@Me-PMA nanocomposite (1)

In order to investigate the catalytic performance of the LDH@Me-PMA nanocomposite (**1**), it was used as a catalyst for the synthesis of 2,3-dihydro quinazoline and dihydropyrimidinones derivatives in the model reaction. Therefore, factors affecting the reaction such as solvent, temperature, amount of catalyst, and time were investigated (Table 1). Therefore, model reaction (**1**) for the synthesis of 2,3-dihydroquinazoline derivatives in the presence of aldehyde (**2**, 0.5 mmol), isatoic anhydride (**3**, 0.5 mmol), ammonium acetate (**4**, 2.5 mmol), and the model reaction (**2**) for the synthesis of 3,4-dihydropyrimidinone-2-(1*H*)-ones in the presence of aldehydes (**2**, 1 mmol), ethyl acetoacetate (**6**, 1 mmol), urea (**7**, 1.2 mmol) were performed to determine the optimal conditions. First, the model reactions were evaluated in the absence of catalyst using different solvents at different temperature conditions (Table 1, **Entries 1–5**). As seen, in the absence of a catalyst, none of the desired products were formed after 2 h. In the following, the reaction in the presence of Me-PMA and Mg–Al LDH as a catalyst was performed, That the desired products were achieved with an efficiency of 40–50 % (Table 1, **Entries 6 and 7**). Also, the model reaction in the presence of LDH@Me-PMA nanocatalyst (**1**) was investigated in different solvents and temperatures, the EtOH solvent under reflux conditions with the highest efficiency was selected as the optimal solvent for the synthesis of derivatives **5a** and **8a** (Table 1, **Entries 8–12**). In order to determine the optimal amount of catalyst, the model reactions were carried out in EtOH solvent under reflux conditions in the presence of 20, 30, 50, and 70 mg of nanocatalyst (**1**) (Table 1, **Entries 13–16**). Hence, 30 mg of catalyst (**1**) were determined as optimal amounts for the synthesis of derivatives 5a and 8b. Therefore, the EtOH solvent under reflux conditions in the presence of 30 mg of catalyst, was determined as the optimal condition for the synthesis of other derivatives (Table 2, Table 3).

**Table 1:** Synthesis of synthesis of 2,3-dihydro quinazoline and 3,4-dihydropyrimidinone-2-(1*H*)-ones derivatives in different conditions^a^,^b^..

Entry	catalyst	Catalyst Loading (mg)	solvent	Time (min)	temperature (°C)	Yield (%)	
						Reaction (1)^(^a^)^	Reaction (2)^(^b^)^
1	–	–	EtOH	120	Reflux	Trace	Trace
2	–	–	H_2_O	120	Reflux	–	–
3	–	–	EtOH/H2O	120	Reflux	–	–
4	–	–	THF	120	Reflux	–	–
5	–	–	ACN	120	Reflux	–	–
6	Me-PMA	30	EtOH	60	Reflux	50	40
7	Mg–Al LDH	30	EtOH	60	Reflux	40	45
8	**LDH@Me-PMA (1)**	**30**	**EtOH**	**20**	**Reflux**	**98**	**97**
9	LDH@Me-PMA (**1**)	30	H_2_O	30	Reflux	65	60
10	LDH@Me-PMA (**1**)	30	EtOH/H_2_O	30	Reflux	70	70
11	LDH@Me-PMA (**1**)	30	THF	30	Reflux	45	43
12	LDH@Me-PMA (**1**)	30	ACN	30	Reflux	50	50
13	LDH@Me-PMA (**1**)	30	EtOH	20	r.t.	57	60
14	LDH@Me-PMA (**1**)	20	EtOH	20	Reflux	80	85
15	LDH@Me-PMA (**1**)	50	EtOH	20	Reflux	75	75
16	LDH@Me-PMA (**1**)	70	EtOH	20	reflux	65	65

a Reaction conditions (**1**): 4-cholorobenzaldehyde (**2**, 0.5 mmol), isatoic anhydride (**3**, 0.5 mmol), ammonium acetate (**4**, 2.5 mmol) in different conditions.

b Reaction conditions (**2**): 4-cholorobenzaldehyde (**2**, 1 mmol), ethyl acetoacetate (**6**, 1 mmol), urea (**7**, 1.2 mmol) in different conditions.

**Table 2:**
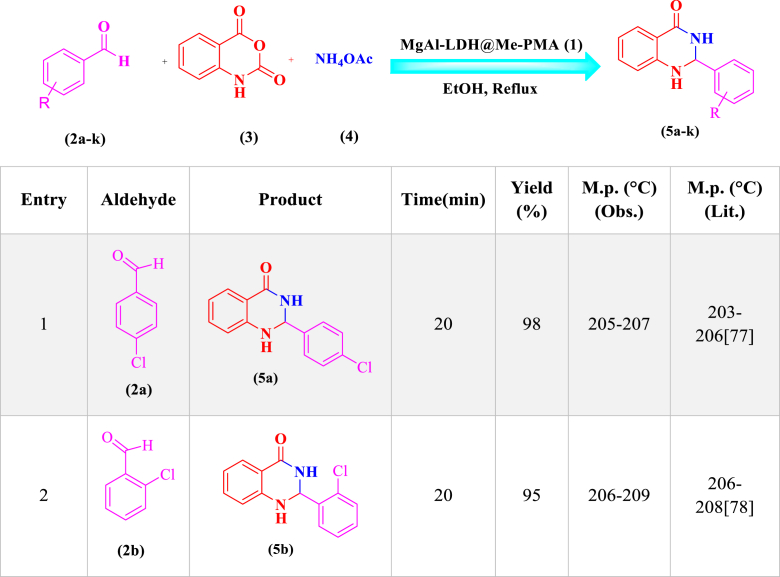

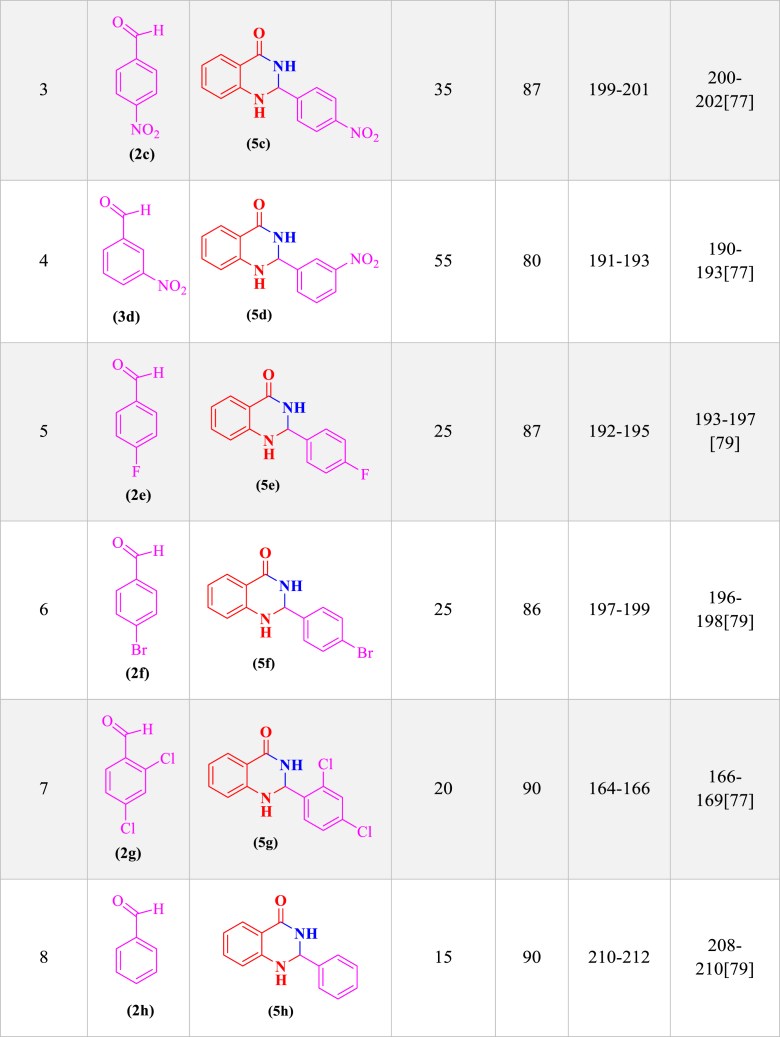

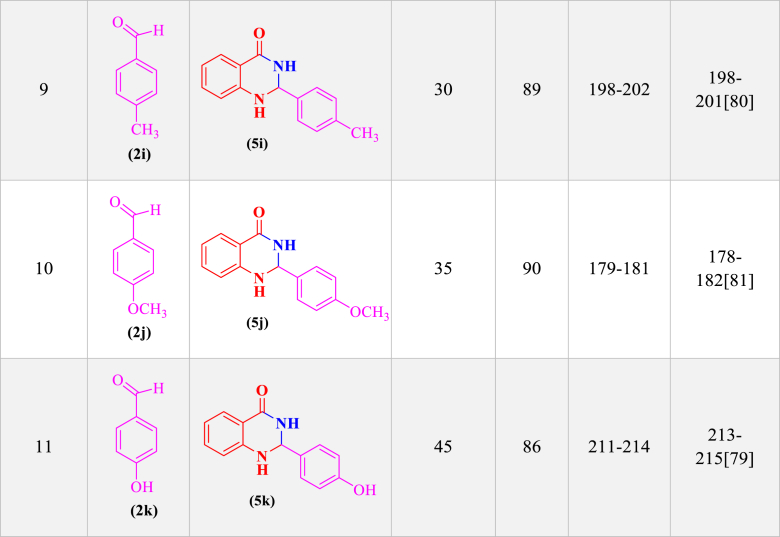
Synthesis of 2,3-dihydroquinazoline derivatives (**5a-k**) through condensation of aldehyde derivatives (**2a-k**), isatoic anhydride (**3**), and ammonium acetate (**4**) in the presence of LDH@Me-PMA (**1**)^a^.

a Reaction conditions: aldehyde derivatives (**2a-k**, 1 mmol), isatoic anhydride (**3**, 1 mmol) and ammonium acetate (**4**, 2.5 mmol) in the presence of LDH@Me-PMA (**1**, 30 mg) in EtOH under reflux conditions.

**Table 3:**
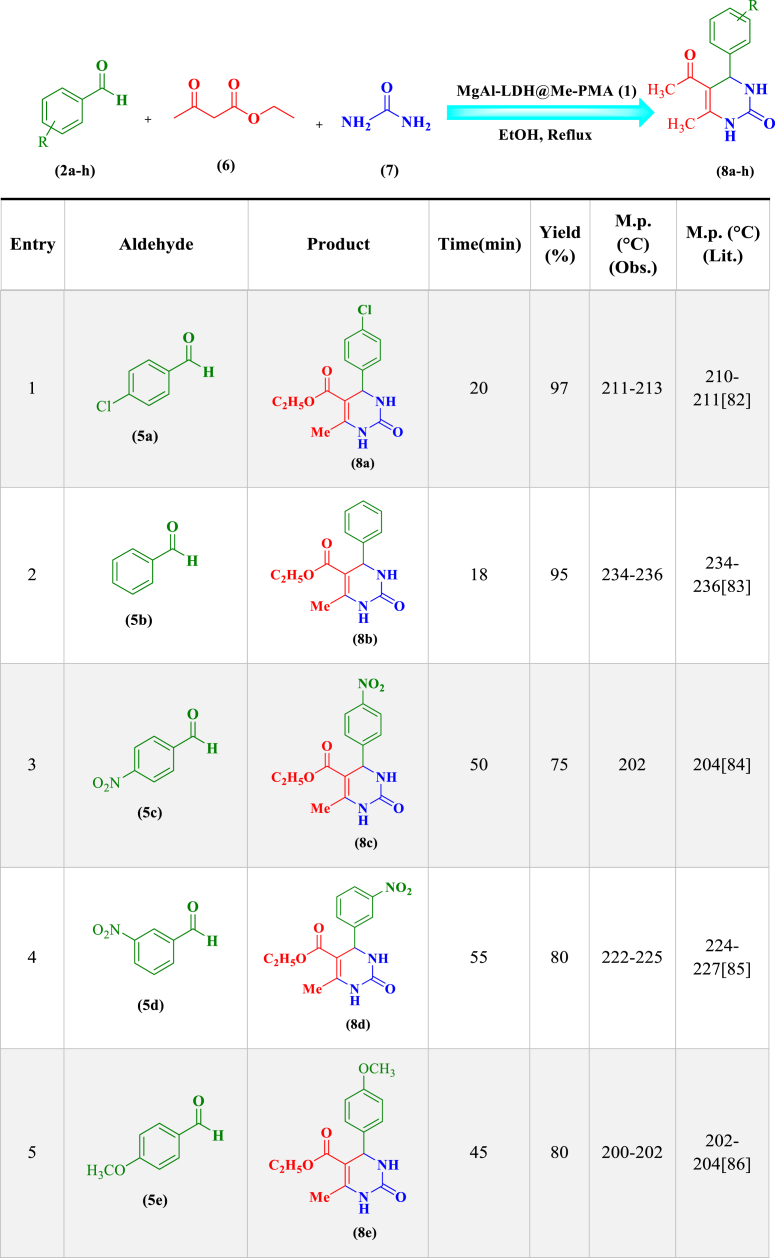

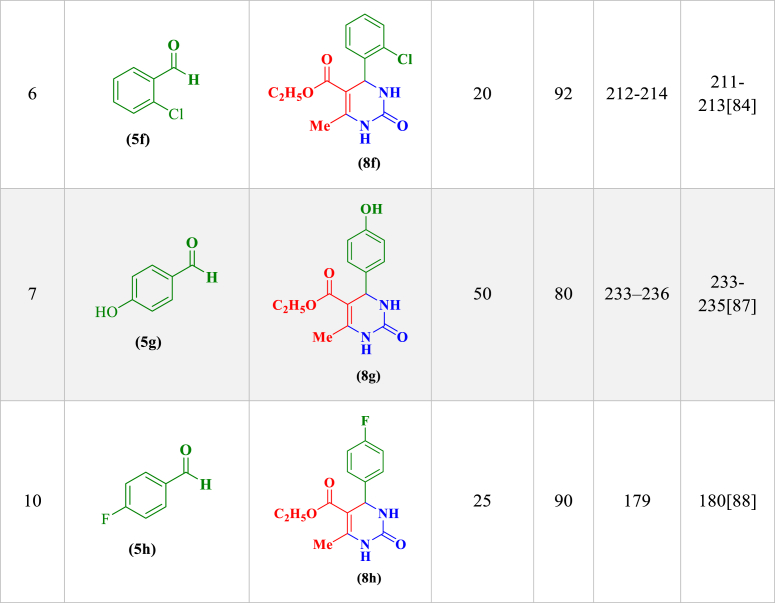
Synthesis of 3,4-dihydropyrimidinone-2-(1*H*)-ones (**8a-h**) through condensation of aldehyde derivatives (**2**, 1 mmol), ethyl acetoacetate (**6**, 1 mmol), urea (**7**, 1.2 mmol) in the presence of LDH@Me-PMA (**1**)^b^.

b Reaction conditions: 4-cholorobenzaldehyde (**2**, 1 mmol), ethyl acetoacetate (**6**, 1 mmol), urea (**7**, 1.2 mmol) in the presence of LDH@Me-PMA (**1**, 30 mg) in EtOH under reflux conditions.

## 5 The proposed mechanism for the synthesis of 2,3-dihydro quinazoline and 3,4-dihydropyrimidinone-2-(1*H*)-ones derivatives in the presence of LDH@Me-PMA (1)

**Scheme 3:**
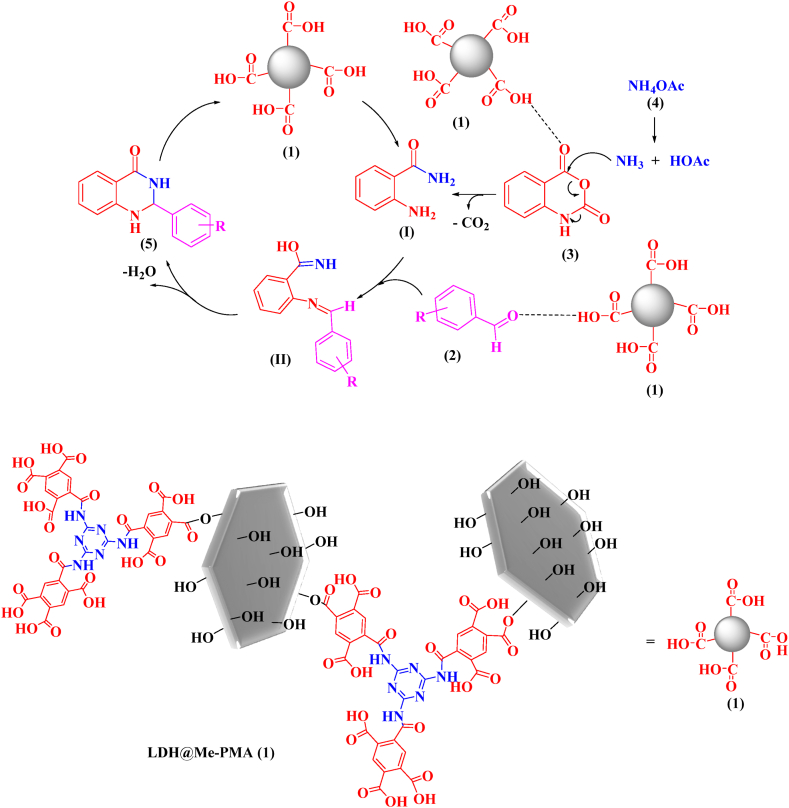
The proposed mechanism for the synthesis of 2,3-dihydroquinazoline derivatives.

The proposed mechanism for the synthesis of 2,3-dihydroquinazoline derivatives is given in Scheme 3. Since the catalyst LDH@Me-PMA (**1**) has acidic properties, it activates the carbonyl groups in the isotonic anhydride for the nucleophilic addition of ammonium acetate (**4**), leading to the formation of intermediate (**I**). Then, by reacting intermediate (**I**) with aldehyde, intermediate (**II**) is formed. In the following, 2,3-dihydroquinazoline derivatives 5 are prepared by removing H_2_O from intermediate (**II**).

**Scheme 4:**
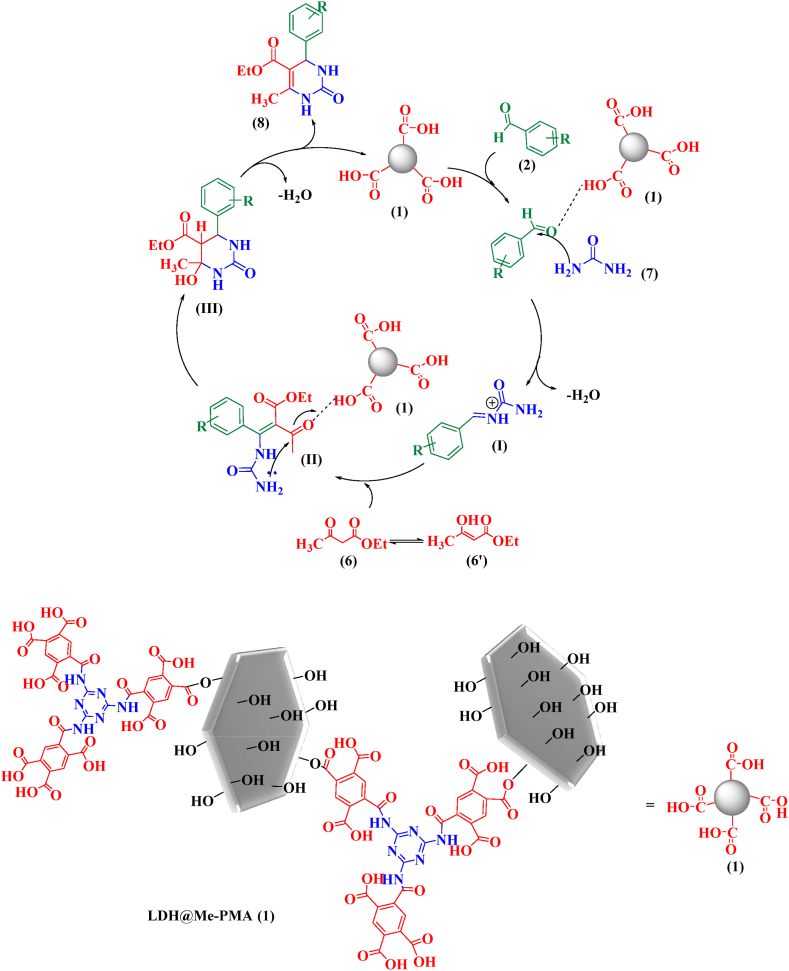
Proposed mechanism for the synthesis of 3,4-dihydropyrimidin-2(1*H*)-ones.

The proposed mechanism for the synthesis of 3,4-dihydropyrimidinone-2-(1*H*)-ones derivatives by LDH@Me-PMA nanocomposite (**1**) is presented in Scheme 4. Initially, the acidic LDH@Me-PMA nanocatalyst (**1**) activates the carbonyl group of aldehyde (**2**) for the nucleophilic addition of urea **7**, leading to the formation of intermediate (**I**) which is an iminium is formed by the loss of a water molecule. In the following, adding the enol form of ethyl acetoacetate (**6**) to intermediate (**I**) leads to the formation of intermediate (**II**). Then, by cyclization of intermediate (**II**), intermediate (**III**) is formed, after which the desired product (**8**) is synthesized by losing a molecule of water.

**Fig. 7:**
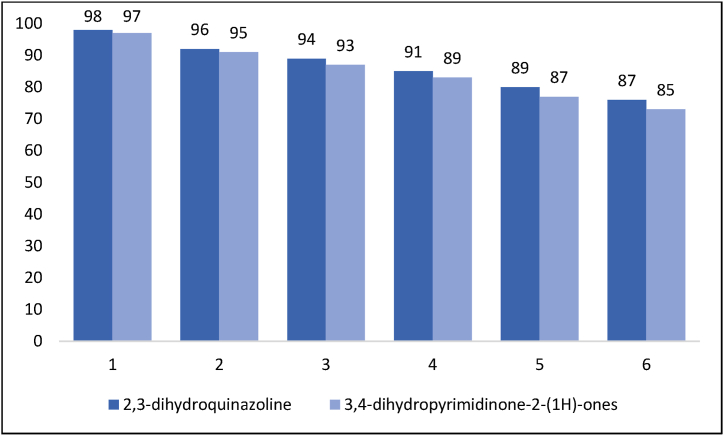
Reusability of the LDH@Me-PMA nanocomposite (**1**) in the model reactions.

**Fig. 8:**
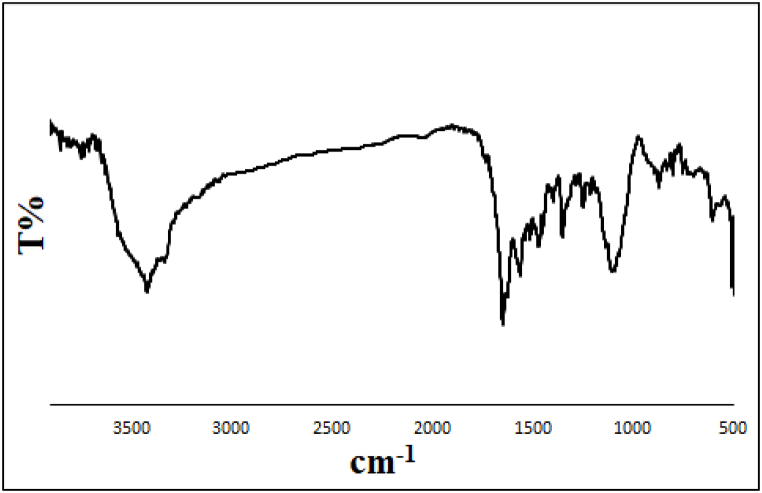
FTIR spectra of recycled LDH@Me-PMA nanocomposite (**1**).

**Fig. 9:**
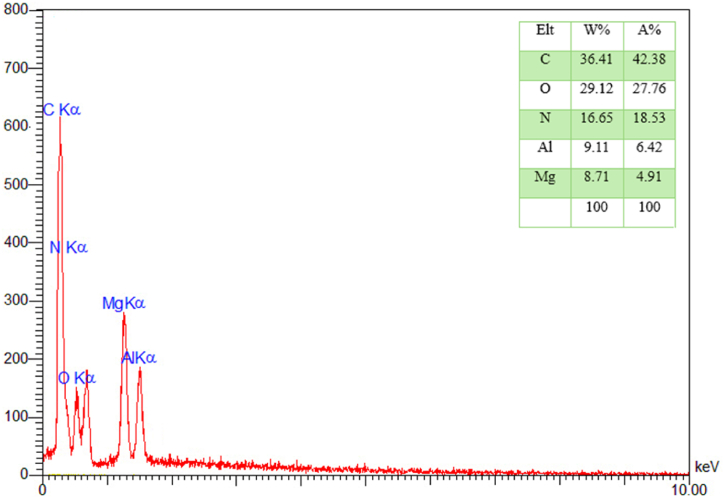
EDX spectra of recycled LDH@Me-PMA nanocomposite (**1**).

Having the ability to recycle and reuse is one of the unique and interesting features of catalysts. Therefore, for this purpose, the nanocatalyst LDH@Me-PMA (**1**) was separated by filtration after the reaction, washed with water and ethanol, and then dried at 80 °C for 12 h. Next, the recovered catalyst was reused in the reaction to prepare 2,3-dihydroquinazoline and 3,4-dihydropyrimidinone-2-(1*H*)-ones derivatives. The results showed that the prepared catalyst has the ability to perform both reactions up to five cycles without significant loss of activity (Fig. 7). Also, FT-IR and EDX analyses performed on the catalyst after the 5th reaction cycle showed no significant change in the initial structure of the LDH@Me-PMA nanocomposite (**1**) (Fig. 8, Fig. 9).

Table 4, Table 5 shows the catalytic efficiency of LDH@Me-PMA nanocomposite (**1**) in comparison with other previously reported catalysts for the synthesis of 2,3-dihydroquinazoline and 3,4-dihydropyrimidinone-2-(1*H*)-ones derivatives. For the purpose of this comparison, various parameters such as catalyst amount, temperature, time, and reaction solvent have been investigated. The results collected in Table 4 show that LDH@Me-PMA catalyst (**1**) has a higher efficiency than previously reported catalysts for the synthesis of 2,3-dihydroquinazoline and 3,4-dihydropyrimidinone-2-(1*H*)-ones derivatives.

**Table 4:** Comparison of catalytic activity of LDH@Me-PMA (**1**) with other reported catalysts for the synthesis of 2,3-dihydro quinazoline derivatives.

Entry	Catalyst	Solvent/Temperature condition	Time (min)	Yield (%)	Reference
1	Wang-OSO_3_H	H_2_O/100 °C	40	84	[77]
2	Montmorillonite-KSF	Solvent-free/100 °C	150	93	[78]
3	Al(H_2_PO_4_)_3_	Solvent-free/100 °C	540	70	[79]
4	Y(NO_3_)_3_.6H_2_O	CH_3_CN	300	97	[80]
**5**	**LDH@Me-PMA (1)**	**EtOH/reflux**	**20**	**98**	**This work**

**Table 5:** Comparison of catalytic activity of LDH@Me-PMA (**1**) with other reported catalysts for the synthesis of 3,4-dihydropyrimidinone-2-(1*H*)-ones derivatives.

Entry	Catalyst	Solvent/Temperature condition	Time (min)	Yield (%)	Reference
1	PANI-FeCl_3_	CH_3_CN/Reflux	1440	83	[81]
2	Zr(H2PO_4_)_2_	Solvent-free/90 °C	60	92	[82]
3	Fe_3_O_4_/PAA-SO_3_H	Solvent-free/RT	120	90	[83]
4	PPF-SO_3_H	EtOH/Reflux	480	81	[84]
**5**	**LDH@Me-PMA (1)**	**EtOH/Reflux**	**20**	**97**	**This work**

## 6 Conclusions

In this work, a heterogeneous acid catalyst was prepared using organic and inorganic components and with the help of melamine G1 dendrimer, and its structure was investigated using various spectral and analytical methods such as FTIR, EDX, XRD, FESEM, BET, and TGA. Relatively good surface area compared to other catalysis with LDH substrate as well as nano particle size has led to the creation of a nanocatalyst with unique properties. After that, LDH@Me-PMA nanocoposite (**1**) was used as an acidic heterogeneous catalyst for the synthesis of 2,3-dihydroquinazoline and 3,4-dihydropyrimidinone-2-(*1H*)-ones derivatives under green conditions. Also, 2,3-dihydroquinazoline and 3,4-dihydropyrimidinone-2-(*1H*)-ones derivatives were prepared under optimal conditions with high to excellent efficiency (98 %). Also, the advantages and characteristics of this method include the ability to recycle and reuse the catalyst for five cycles, the use of cheap and available materials, short reaction time, low catalyst loading, high thermal stability, and the use of green methods and solvents.

## Data availability statement

Data will be made available on request.

## Declaration of competing interest

The authors declare that they have no known competing financial interests or personal relationships that could have appeared to influence the work reported in this paper.
