# Supplementary material for: Pyromellitic acid grafted to cross-linked LDH by dendritic units: An efficient and recyclable heterogeneous catalyst for green synthesis of 2,3-dihydro quinazoline and dihydropyrimidinones derivatives
Source: Heliyon. 2023 Oct 17;9(11):e20978. doi: 10.1016/j.heliyon.2023.e20978 (PMC10623181; doi:10.1016/j.heliyon.2023.e20978)
Supplement: Multimedia component 1 [file mmc1.docx]

**Pyromellitic acid grafted to cross-linked LDH by dendritic units: An efficient and recyclable heterogeneous catalyst for green synthesis of 2,3-dihydro quinazoline and dihydropyrimidinones derivatives**

Nastaran Ghanbari ^a^, Hossein Ghafuri *^a^

*^a^Catalysts and Organic Synthesis Research Laboratory, Department of Chemistry, Iran University of Science and Technology, Tehran 16846‑13114, Iran*

** E-mail:* [ghafuri@iust.ac.ir](mailto:ghafuri@iust.ac.ir)


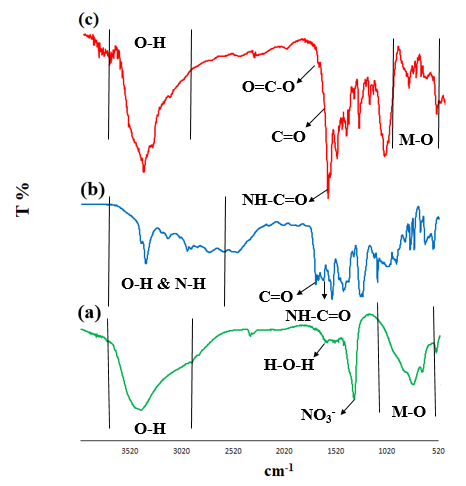


**Figure S1.** FTIR spectra of Mg-Al LDH (**a**), Me-PMA (**b**), and LDH@Me-PMA (**1**, **c**).


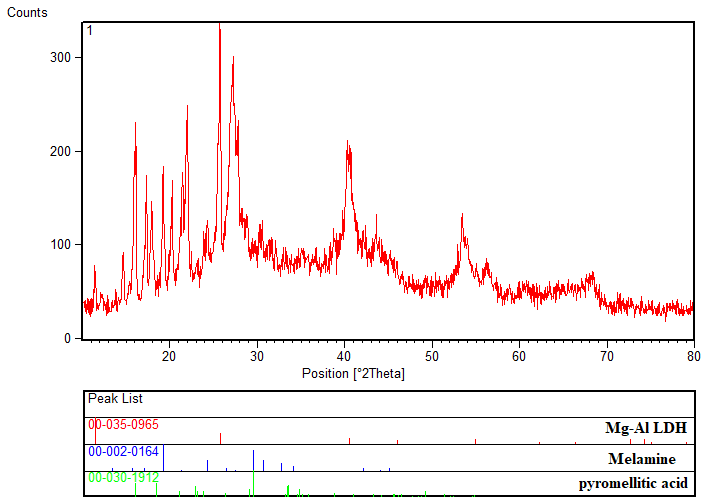


**Figure S2.** XRD patterns of LDH@Me-PMA nanocomposite (**1**).


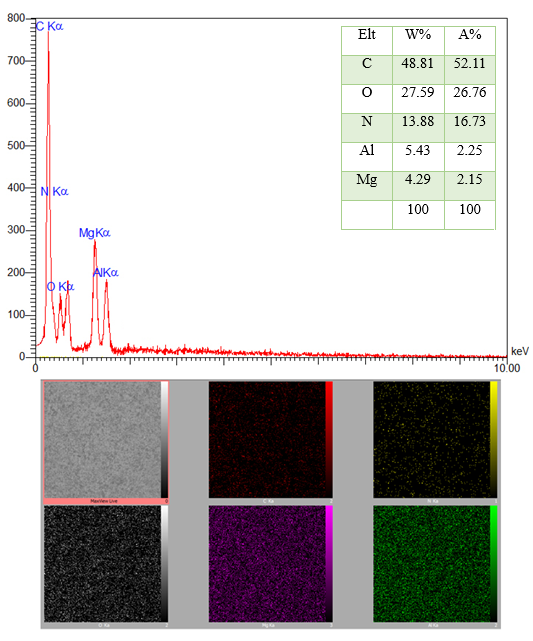


**Figure S3.** EDX spectra of LDH@Me-PMA nanocomposite (**1**).

| 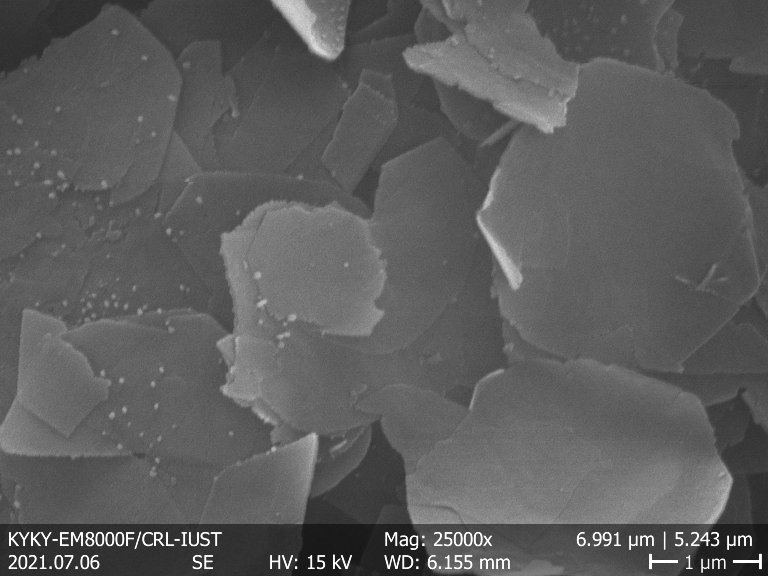  **(a)** | 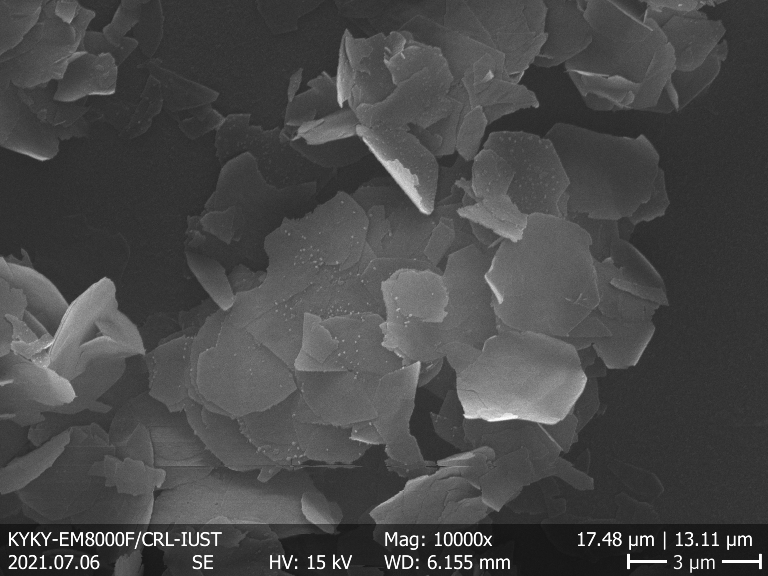  **(b)** |
| --- | --- |
| 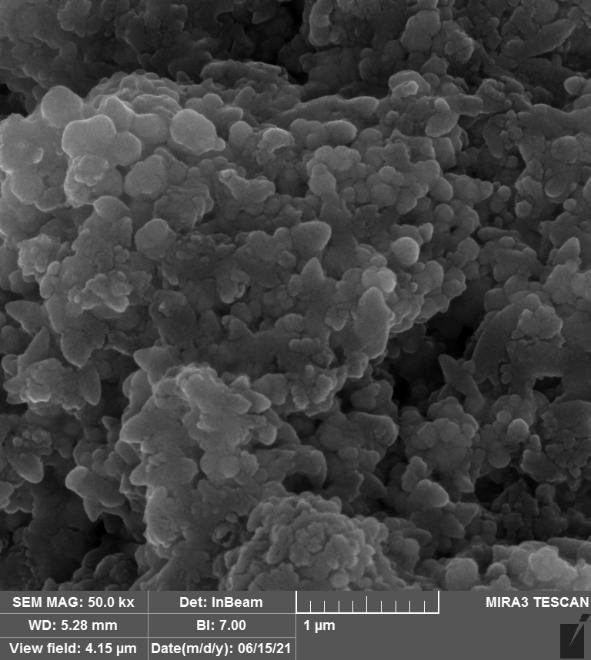  **(c)** | 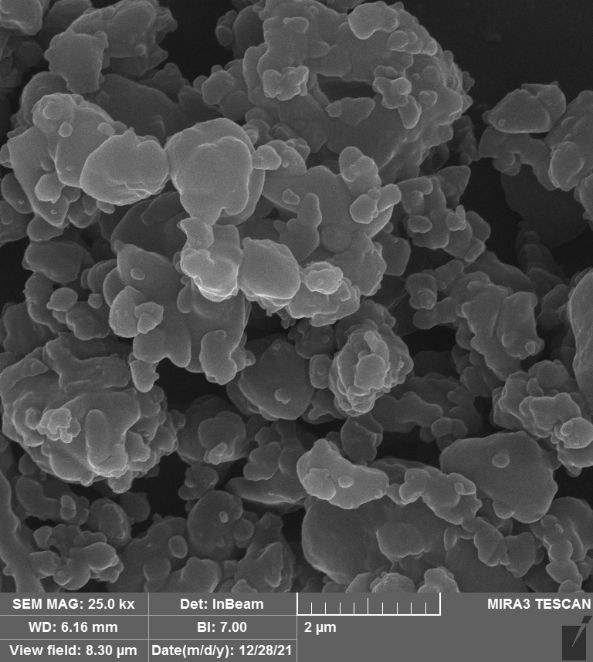  **(d)** |
| 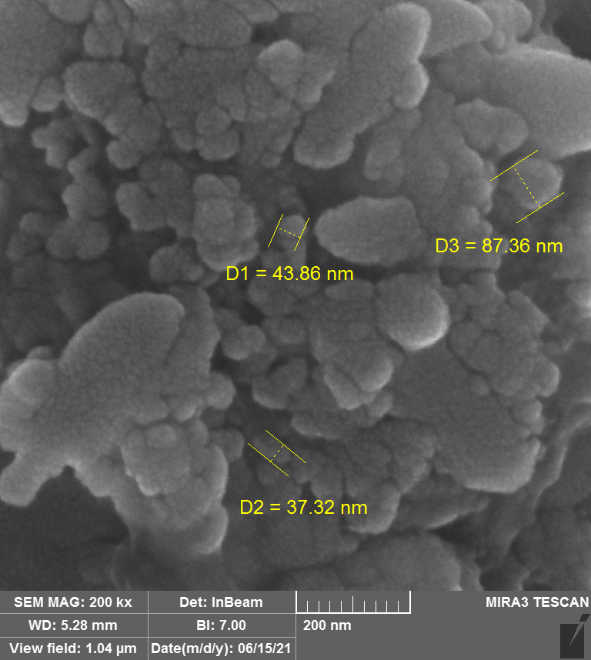  **(e)** | 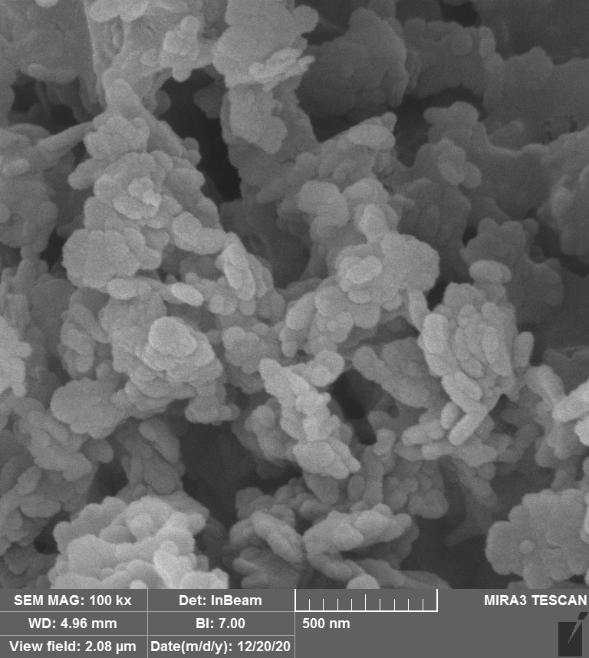  **(f)** |

**Figure S4.** FESEM images of MgAl-LDH (**a** and **b**) and LDH@Me-PMA nanocomposites (**1, c-f**).
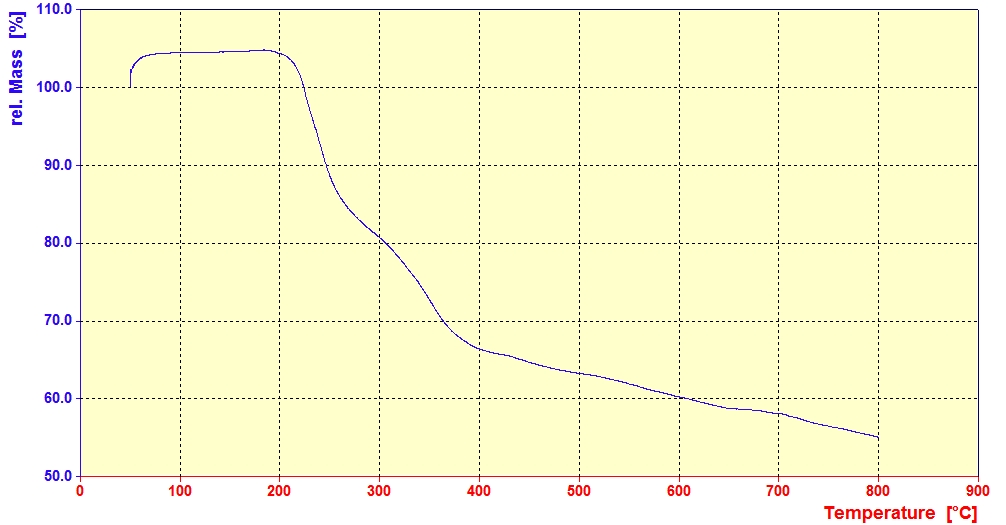


**Figure S5.** TGA curve of the LDH@Me-PMA nanocomposite (**1**).

| 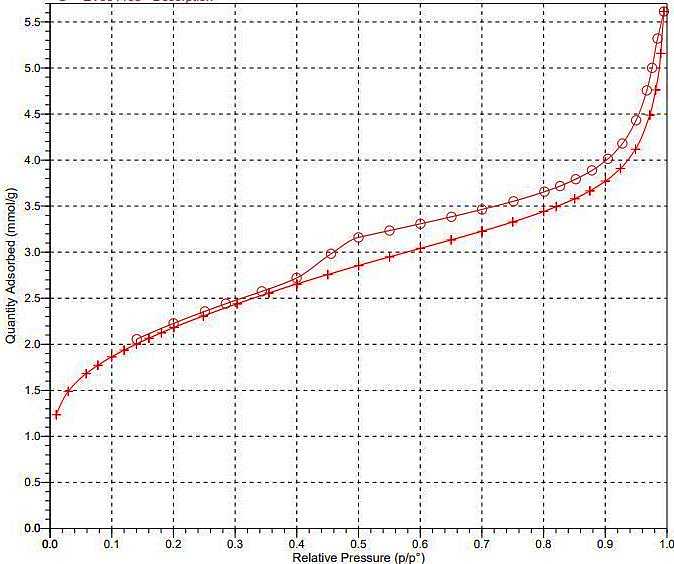 | 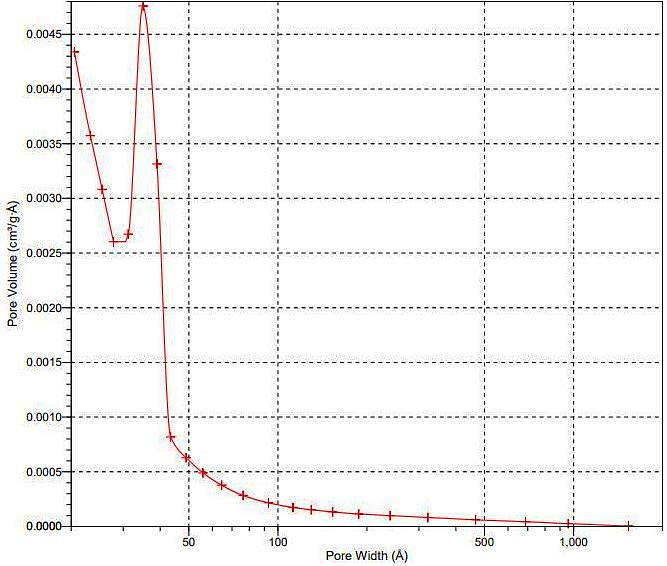 |
| --- | --- |

**Figure S6**. N_2_ adsorption–desorption isotherms of LDH@Me-PMA nanocomposite (**1**),

**Table S4**. Comparison of catalytic activity of LDH@Me-PMA (**1**) with other reported catalysts for the synthesis of 2,3-dihydro quinazoline derivatives.

| **Entry** | **Catalyst** | **Solvent/Temperature condition** | **Time (min)** | **Yield (%)** | **Reference** |
| --- | --- | --- | --- | --- | --- |
| 1 | Wang-OSO_3_H | H_2_O/100 ˚C | 40 | 84 | [1] |
| 2 | Montmorillonite-KSF | Solvent-free/100 ˚C | 150 | 93 | [2] |
| 3 | Al(H_2_PO_4_)_3_ | Solvent-free/100 ˚C | 540 | 70 | [3] |
| 4 | Y(NO_3_)_3_.6H_2_O | CH_3_CN | 300 | 97 | [4] |
| **5** | **LDH@Me-PMA (1)** | **EtOH/reflux** | **20** | **98** | **This work** |

**Table S5**. Comparison of catalytic activity of LDH@Me-PMA (**1**) with other reported catalysts for the synthesis of 3,4‑dihydropyrimidinone‑2‑(1*H*)‑ones derivatives.

| **Entry** | **Catalyst** | **Solvent/Temperature condition** | **Time (min)** | **Yield (%)** | **Reference** |
| --- | --- | --- | --- | --- | --- |
| 1 | PANI-FeCl_3_ | CH_3_CN / Reflux | 1440 | 83 | [5] |
| 2 | Zr(H2PO_4_)_2_ | Solvent-free/90 ˚C | 60 | 92 | [6] |
| 3 | Fe_3_O_4_/PAA-SO_3_H | Solvent-free/RT | 120 | 90 | [7] |
| 4 | PPF-SO_3_H | EtOH/Reflux | 480 | 81 | [8] |
| **5** | **LDH@Me-PMA (1)** | **EtOH/Reflux** | **20** | **97** | **This work** |

**Selected spectral data**

2-(4-chloro-phenyl)-2,3‑dihydroquinazolin‑4(1H)‑one

FTIR (KBr, cm^-1^): 3305, 3184, 3062, 1654, 1606, 1431, 1090, 749 cm^-1^. ^1^H NMR (500 MHz, DMSO): δ H (ppm)= 5.77(s, 1H, CH), 6.68(t, 1H, Ar-H), 6.74(d, 1H, Ar-H), 7,1(s, 1H, NH), 7.24(t, 1H, Ar-H), 7.45(d, 1H, Ar-H), 7.50(d, 1H, Ar-H), 7.61(d, 1H, Ar-H), 8.27(s, 1H, CONH).


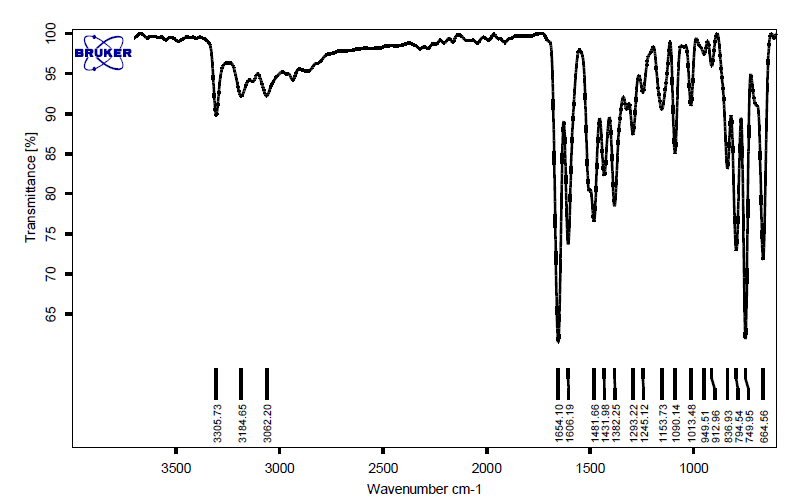


**Figure S7.** FTIR spectrum of the 2-(4-chloro-phenyl)-2,3‑dihydroquinazolin‑4(1H)‑one.


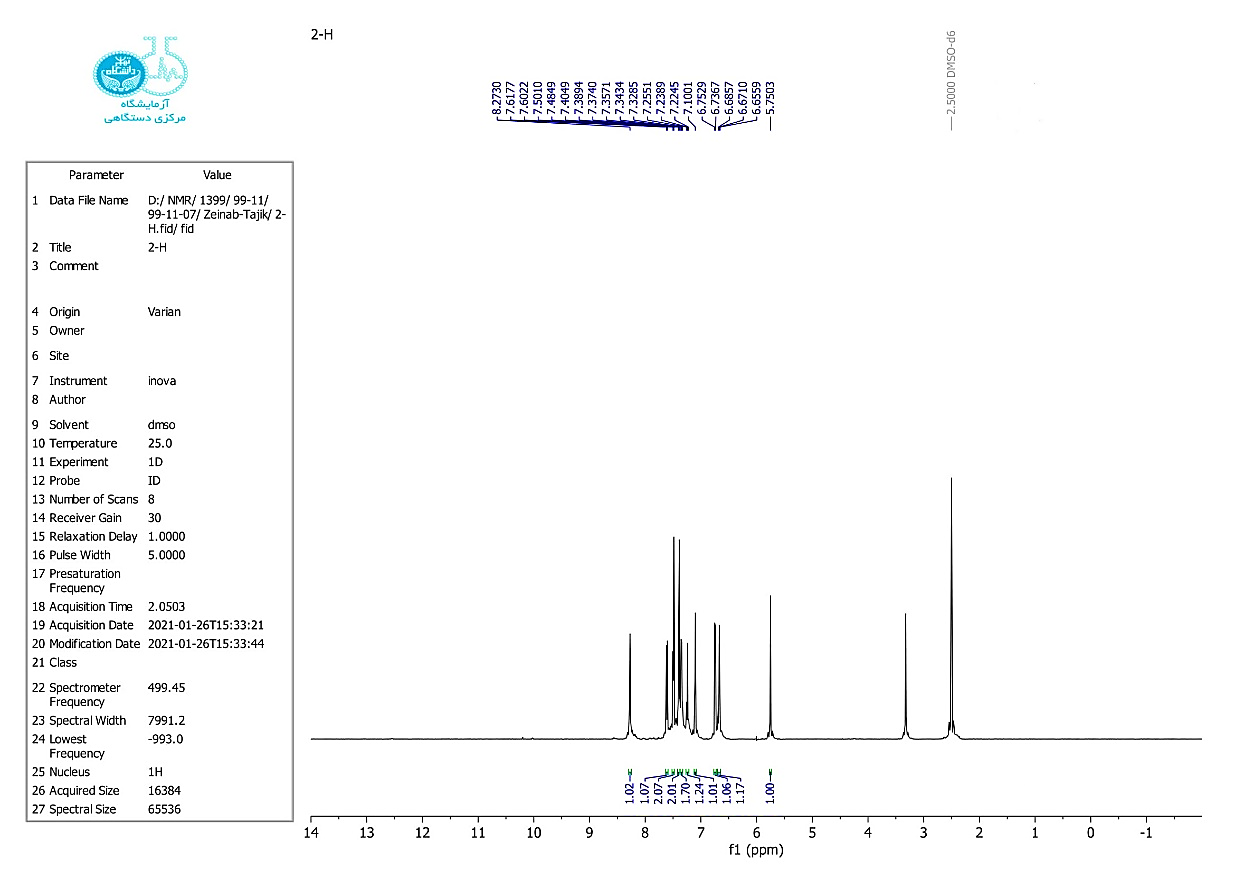


**Figure S8.** 1HNMR spectrum of the 2-(4-chloro-phenyl)-2,3‑dihydroquinazolin‑4(1H)‑one.

2‑(2‑Chlorophenyl)‑2,3‑dihydroquinazolin‑4(1H)‑one

Mp: 206– 209 °C. ^1^HNMR (500 MHz, DMSO-d6): δ (ppm) 6.15 (s, 1H), 6.74 (t, 1H, *J* = 7.4 Hz), 6.76 (d, 1H, *J* = 8.2 Hz), 7.03 (s, 1H), 7.25 (t, 1H, *J* = 7.6 Hz), 7.42 (d, 2H, *J* =4.2 Hz), 7.50 (d, 1H, *J* = 4.2 Hz), 7.67 (d, 2H, *J* = 6.71, Hz), 8.23(s, 1H); IR (KBr) max (cm^-1^): 3306, 3170, 3035, 1655, 1486, 1480.

**Figure S9.** FTIR spectra of 2‑(2‑Chlorophenyl)‑2,3‑dihydroquinazolin‑4(1*H*)‑one (**5b**)**.**

**
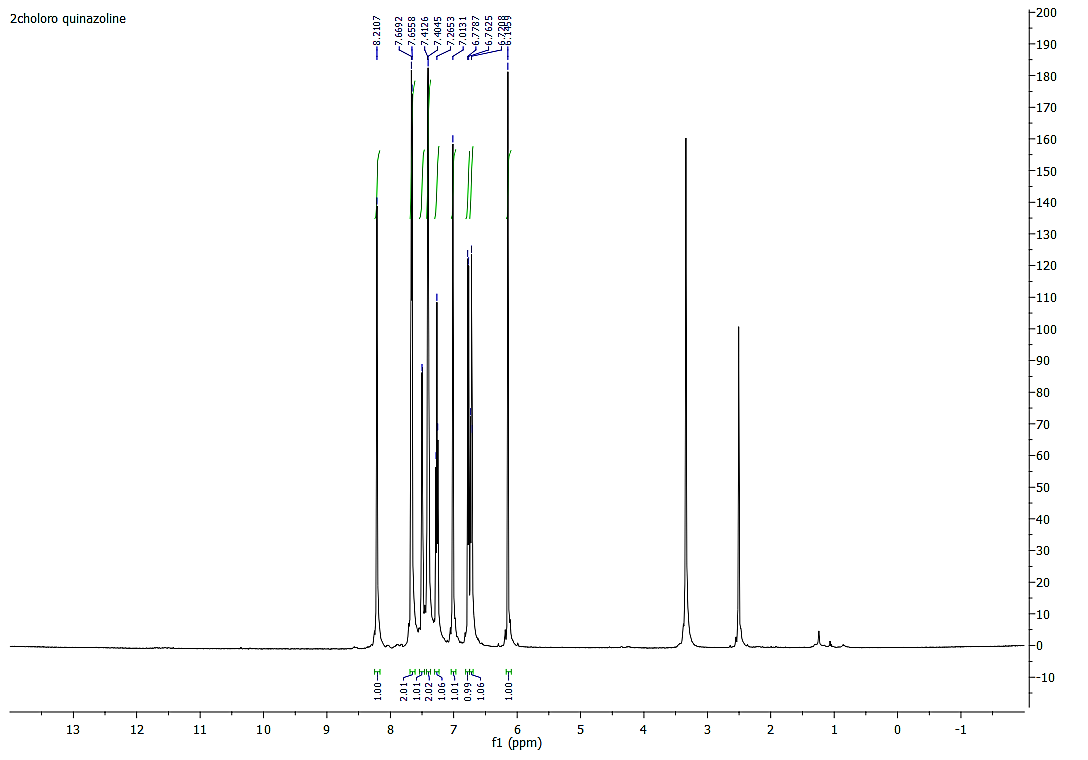
**

**Figure S10.** HNMR of 2‑(2‑Chlorophenyl)‑2,3‑dihydroquinazolin‑4(1H)‑one (**5b**).

2‑(4‑Methylphenyl)‑2,3‑dihydroquinazolin‑4(1H)‑one

Mp: 198-202 °C. ^1^H NMR (500 MHz, DMSO-d6): δ (ppm) 2.30 (s, 3H), 5.72 (s, 1H), 6.65 (t, 1H, *J* = 7.3 Hz), 6.74 (d, 1H, *J* = 8.2 Hz), 7.05 (s, 1H), 7.20 (d, *J* = 7.7 Hz, 1H), 7.24 (t, *J* = 8.2 Hz, 1H), 7.36 (d, *J* = 7.7 Hz, 2H), 7.62 (d, *J* = 7.5 Hz, 1H), 8.23 (s, 1H); IR (KBr) max (cm^-1^): 3291, 3165, 3025, 2930, 2820, 1252.

**Figure S11.** FTIR spectra of 2‑(4‑Methylphenyl)‑2,3‑dihydroquinazolin‑4(1H)‑one **(5i).**

**
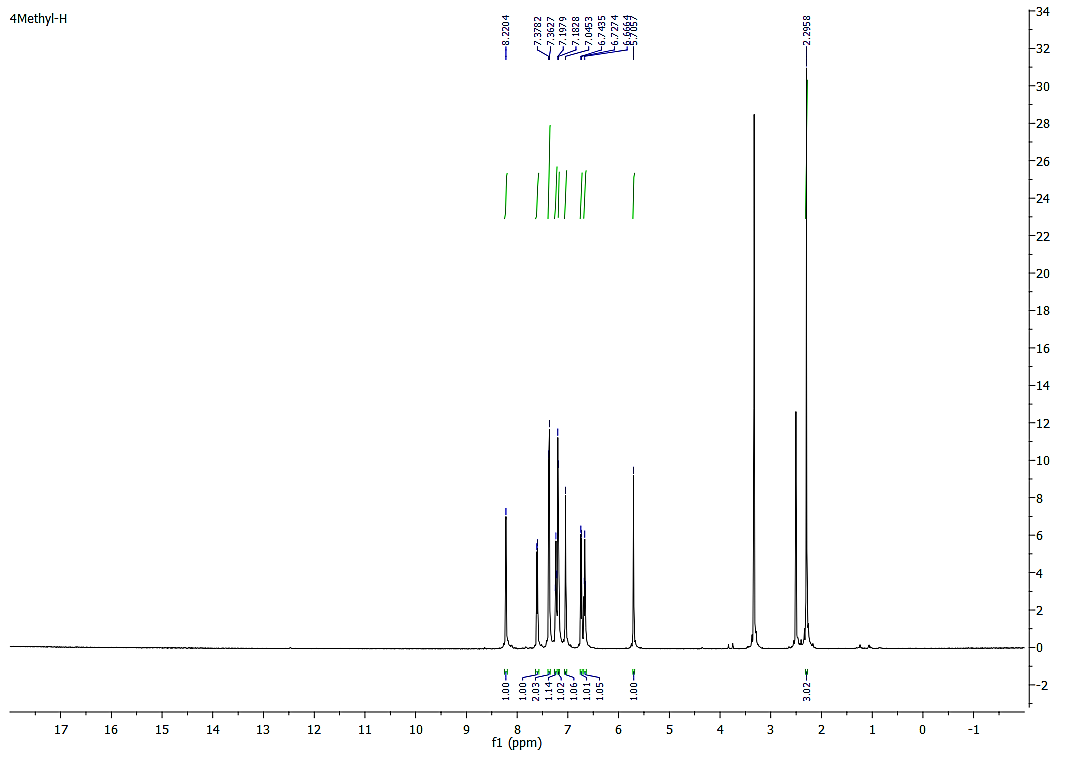
**

**Figure S12.** HNMR of 2‑(4‑Methylphenyl)‑2,3‑dihydroquinazolin‑4(1H)‑one **(5i).**

*4-(2Chloro-phenyl)-6-methyl-2-oxo-1,2,3,4-tetrahydropyrimidine-5-carboxylicacid ethyl ester*

Mp: 212-214 °C. ^1^H NMR (500 MHz, DMSO-d6): δ (ppm) 1.0 (t, 3H, J = 6.9 Hz). 2.31 (s, 3H), 3.90 (q, 2H, J = 7 Hz), 5.64 (d, 1H, J = 2.5 Hz), 7.26–7.42 (m, 4H), 7.70 (s, 1H), 9.27 (s, 1H). IR (KBr) max (cm^-1^): 3355, 3224, 3109, 2979, 1696, 1640, 1452, 1367, 1232, 1099.

**Figure. S13.** FT-IR spectrum *of 4-(2Chloro-phenyl)-6-methyl-2-oxo-1,2,3,4-tetrahydropyrimidine-5-carboxylicacid ethyl ester*

***
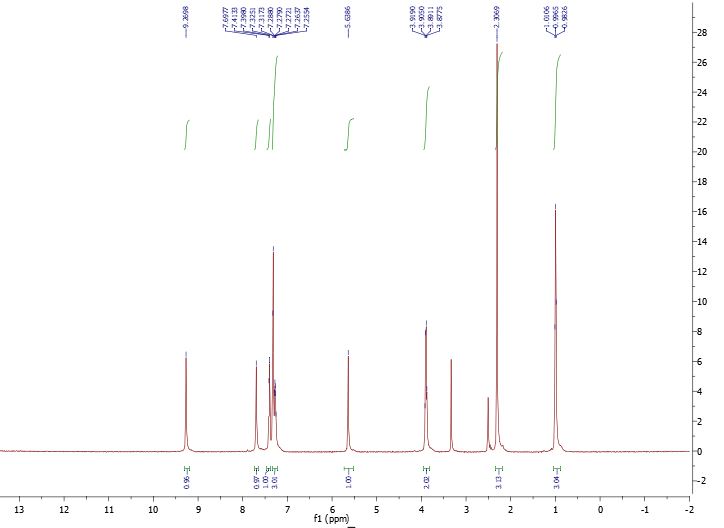
***

**Figure. S7.** ^1^H NMR spectrum of *4-(2Chloro-phenyl)-6-methyl-2-oxo-1,2,3,4-tetrahydropyrimidine-5-carboxylicacid ethyl ester*

*4-(4-Methoxy-phenyl)-6-methyl-2-oxo-1,2,3,4-tetrahydropyrimidine-5-carboxylicacid ethyl ester*

Mp: 200-202 °C. ^1^H NMR (500 MHz, DMSO-d6): δ (ppm) 1.13 (t, J = 6.9 Hz, 3H), 2.25 (s, 3H), 3.73 (s, 3H), 4.20 (q, 2H, J = 7.1 Hz), 5.12 (d, 1H, J = 2.9 Hz), 6.89 (d, 2H, J = 8.5 Hz), 7.17 (d, J = 8.4 Hz), 7.67 (s, 1H) and 9.17 (s, 1H). IR (KBr): max (cm^-1^): 3225, 3097, 2930, 2835, 1713, 1656, 1513.

**Figure. S14.** FT-IR spectrum *of 4-(4-Methoxy-phenyl)-6-methyl-2-oxo-1,2,3,4-tetrahydropyrimidine-5-carboxylicacid ethyl ester*

*
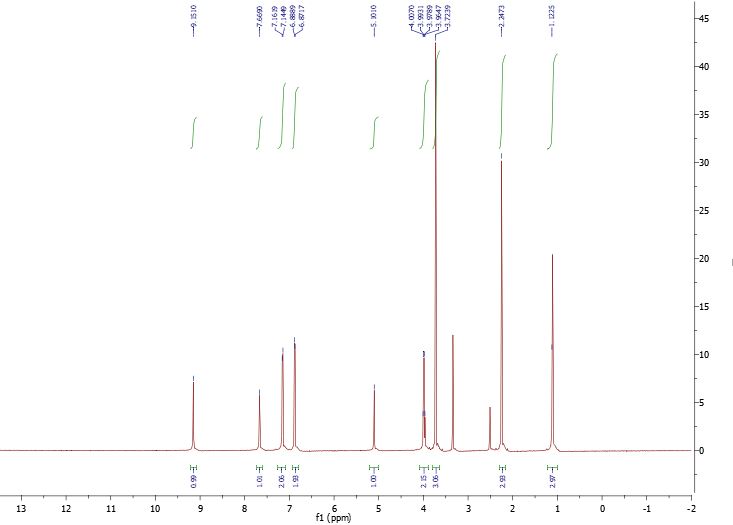
*

**Figure. S15.** ^1^H NMR spectrum *of 4-(4-Methoxy-phenyl)-6-methyl-2-oxo-1,2,3,4-tetrahydropyrimidine-5-carboxylicacid ethyl ester.*

*4-(3-nitrophenyl)- 6-methyl-2-oxo-1,2,3,4-tetrahydropyrimidine-5-carboxylicacid ethyl ester*

Mp: 222-225 ^o^C ; IR (KBr): ν 3322, 3090, 2919, 1690, 1650, 1520 cm^-1^; ^1^H-NMR (500 MHz, DMSO-d6): δ= 1.10 (t, J= 7 Hz, 3H, CH_2_CH_3_), 2.28 (s, 3H, CH_3_), 4 (q, 2H, OCH_2_CH_3_), 5.31 (s, 1H, CH-Ar), 7.66 (t, J= 7.8 Hz, 1H, Ar-H), 7.70 (d,1H, Ar-H), 7.90 (s, 1H, NH), 8.09 (s, 1H, Ar-H), 8.14 (d, 1H, Ar-H), 9.37 (s, 1H, NH).


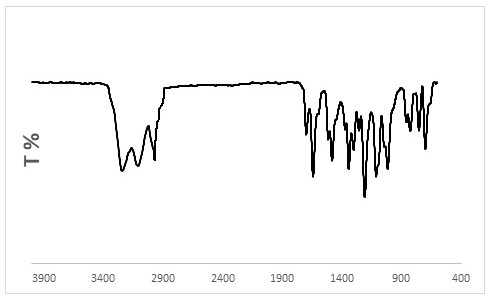


**Figure. S16.** FT-IR spectrum *of 4-(3-nitrophenyl)- 6-methyl-2-oxo-1,2,3,4-tetrahydropyrimidine-5-carboxylicacid ethyl ester*

*
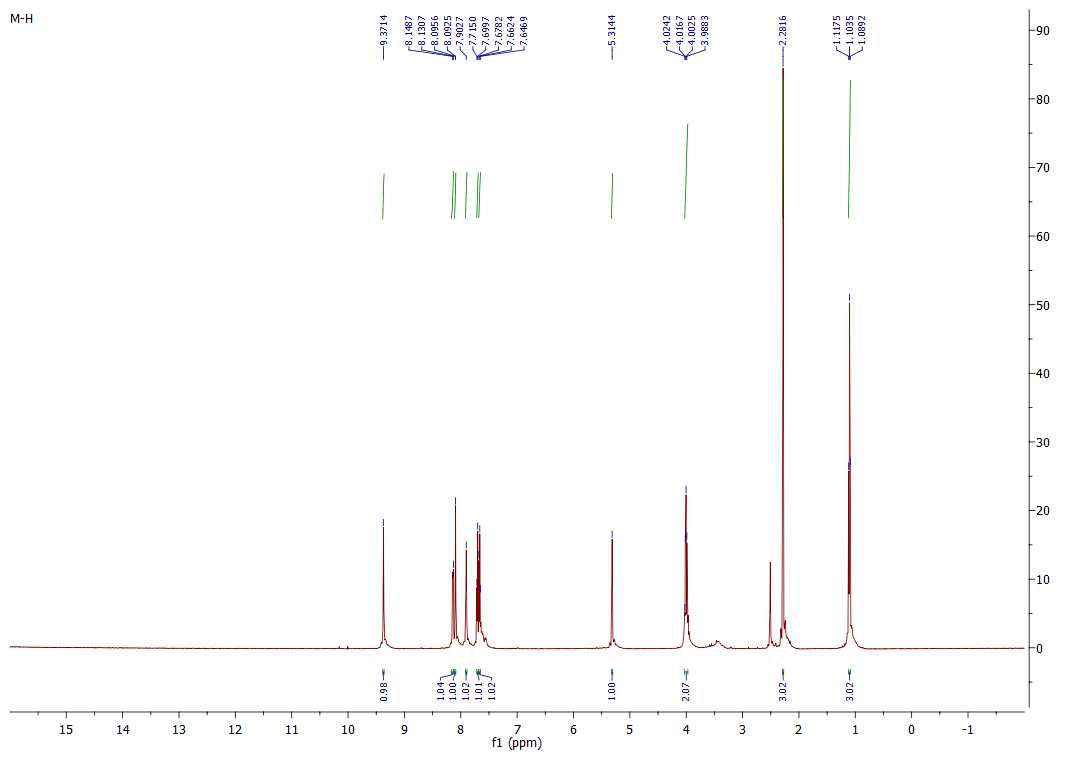
*

**Figure. S17.** ^1^H NMR spectrum *of 4-(3-nitrophenyl)- 6-methyl-2-oxo-1,2,3,4-tetrahydropyrimidine-5-carboxylicacid ethyl ester.*

[1] A.D. Rao, B. Vykunteswararao, T. Bhaskarkumar, N.R. Jogdand, D. Kalita, J.K.D. Lilakar, V. Siddaiah, P.D. Sanasi, A. Raghunadh, Sulfonic acid functionalized Wang resin (Wang-OSO3H) as polymeric acidic catalyst for the eco-friendly synthesis of 2, 3-dihydroquinazolin-4 (1H)-ones, Tetrahedron letters, 56 (2015) 4714-4717.

[2] S.U. Tekale, S.B. Munde, S.S. Kauthale, R.P. Pawar, An efficient, convenient, and solvent-free synthesis of 2, 3-dihydroquinazolin-4 (1 H)-ones using montmorillonite-KSF clay as a heterogeneous catalyst, Organic Preparations and Procedures International, 50 (2018) 314-322.

[3] H.R. Shaterian, A.R. Oveisi, M. Honarmand, Synthesis of 2, 3-dihydroquinazoline-4 (1 H)-ones, Synthetic Communications®, 40 (2010) 1231-1242.

[4] A.A. Khan, K. Mitra, A. Mandal, N. Baildya, M.A. Mondal, Yttrium nitrate catalyzed synthesis, photophysical study, and TD‐DFT calculation of 2, 3‐dihydroquinazolin‐4 (1H)‐ones, Heteroatom Chemistry, 28 (2017) e21379.

[5] F. Zamani, E. Izadi, Synthesis and characterization of sulfonated-phenylacetic acid coated Fe3O4 nanoparticles as a novel acid magnetic catalyst for Biginelli reaction, Catalysis Communications, 42 (2013) 104-108.

[6] M. KÜÇÜKİSLAMOĞLU, Ş. BEŞOLUK, M. Zengin, M. Arslan, M. Nebioğlu, An efficient one-pot synthesis of dihydropyrimidinones catalyzed by zirconium hydrogen phosphate under solvent-free conditions, Turkish Journal of Chemistry, 34 (2010) 411-416.

[7] P. Salehi, M. Dabiri, M.A. Zolfigol, Efficient synthesis of 3, 4-dihydropyrimidin-2 (1H)-ones over silica sulfuric acid as a reusable catalyst under solvent-free conditions, Heterocycles, 60 (2003) 2435-2440.

[8] H.A. Patel, A.M. Sawant, V.J. Rao, A.L. Patel, A.V. Bedekar, Polyaniline supported FeCl 3: an effective heterogeneous catalyst for Biginelli reaction, Catalysis Letters, 147 (2017) 2306-2312.
